# Fissured Tongue among Patients Visiting the Department of Oral Medicine and Radiology in a Tertiary Care Centre

**DOI:** 10.31729/jnma.8287

**Published:** 2023-10-31

**Authors:** Harleen Bali, Sagar Adhikari, Chandan Upadhyaya, Pratibha Poudel, Bhoj Raj Adhikari, Sanju Pandit, Sneha Sapkota

**Affiliations:** 1 Department of Oral Medicine and Radiology, Kathmandu University School of Medical Sciences, Dhulikhel, Kavrepalanchok, Nepal; 2 Department of Oral and Maxillofacial Surgery, Kathmandu University School of Medical Sciences, Dhulikhel, Kavrepalanchok, Nepal; 3 Department of Oral and Maxillofacial Pathology, Kathmandu University School of Medical Sciences, Dhulikhel, Kavrepalanchok, Nepal

**Keywords:** *anatomic variation*, *fissured tongue*, *Nepal*, *oral mucosa*, *prevalence*

## Abstract

**Introduction:**

Examination of the tongue and knowledge of its variation can aid clinicians in correctly assessing the cause of a patient's complaint. Despite World Health Organization recommendations to encourage more epidemiological assessment of oral mucosal variations and lesions, the volume of literature in this area is limited. This study aimed to find out the prevalence of fissured tongues among patients visiting the Department of Oral Medicine and Radiology in a tertiary care centre.

**Methods:**

A descriptive cross-sectional study was conducted among patients visiting the Department of Oral Medicine and Radiology in a tertiary care centre. Data was collected from 12 March 2023 to 10 May 2023 after obtaining ethical approval from the same institute. A convenience sampling method was used. The point estimate was calculated at a 95% Confidence Interval.

**Results:**

Out of 540 patients, the prevalence of fissured tongue was 73 (13.51%) (10.63-16.40, 95% Confidence Interval). A total of 34 (46.57%) were males and 39 (53.42%) were females.

**Conclusions:**

The prevalence of fissured tongue among patients visiting the Department of Oral Medicine and Radiology was higher than other studies done in similar settings.

## INTRODUCTION

One of the common manifestations of tongue is a fissured tongue, scrotal tongue or lingua plicata. It manifests as a deep groove on the dorsum surface of the tongue which can vary in number, depth and orientations. The grooves can act as retention areas for food and microbes leading to inflammation which is experienced by patients as pain, burning sensation and malador.^12^

Previous studies have revealed that a fissured tongue and its pathologies are related to genetics, fungal infections, the use of smokeless tobacco and certain systemic diseases including hypertension, psoriasis, orofacial granulomatosis and diabetes mellitus.^1^ Prevalence and the extent of clinical appearance of dental anomalies are varied across geographical areas. Despite World Health Organization (WHO) recommendations to encourage more epidemiological assessment of oral mucosal variations and lesions, the volume of literature in this area is limited.^2^

This study aimed to find out the prevalence of fissured tongue among patients visiting the Department of Oral Medicine and Radiology in a tertiary care centre.

## METHODS

A descriptive cross-sectional study was conducted at Dhulikhel Hospital, Dhulikhel, Kavrepalanchok, Nepal from 12 March to 10 May 2023. Ethical approval was obtained from the Institutional Review Committee (Reference number: 32/23). All the patients visiting the Department of Oral Medicine and Radiology, willing to participate in the study were included after obtaining written informed consent. Patients with difficulty in opening their mouths and with dental emergencies (such as space infections, trauma, dyspnea, and dysphagia) were excluded from the study. A convenience sampling method was used. The sample size was calculated using the following formula:


$$\begin{array}{ll} \text{n} & ={\text{Z}}^{2}\times \frac{\text{p}\times \text{q}}{{\text{e}}^{\text{2}}}\\ & =1.96^{2}\times \frac{0.50\times 0.50}{0.05^{2}}\\ & =483 \end{array}$$

Where,

n = minimum required sample size
Z = 1.96 at 95% of Confidence Interval (CI)
p = prevalence taken from a previous study, 13%^3^
q = 1-p
e = margin of error, 3%

The calculated minimum sample size was 483. However, 540 participants were included in the study.

Subjects were examined in dental chairs. The oral examination was done under chair light. The tongue was screened for any variation of fissures. Instruments for oral examination used were: plane mouth mirrors; metallic periodontal probes (Community Periodontal Index (CPI) probe) that confirm WHO specifications, i.e. 0.5 mm ball tip; a black band between 3.5 and 5.5 mm and rings at 8.5 and 11.5 mm from the ball tip; and pair of tweezers; pair of disposable gloves; gauge.^2^ The fissures pattern was noted.^4^

The data obtained was entered and analyzed using the IBM SPSS Statistics version 20.0. The point estimate was calculated at a 95% CI.

## RESULTS

**Figure 1:**
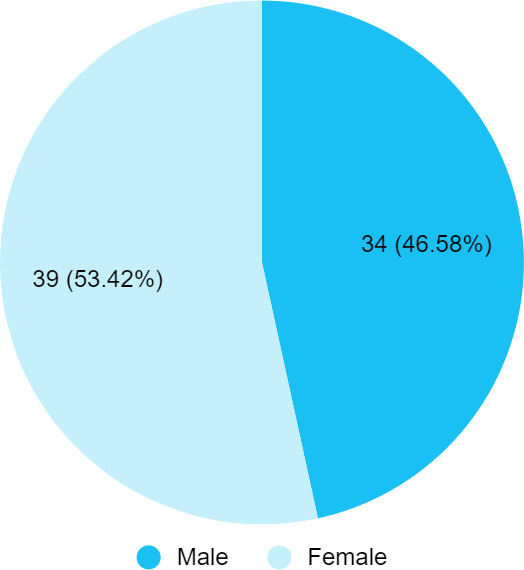
Sex-wise distribution of patients with fissured tongue (n= 73).

Among 540 patients, the fissured tongue was present in 73 (13.51%) (10.63-16.40, 95% CI) participants. The age range of participants was 16-80 years with a mean age of 31.74±7.26 years. About 39 (53.42%) were females with a female-to-male ratio of 1.15. A total of 37 (50.68%) had a single prominent median groove, 16 (21.91%) had a median groove with accessory grooves radiating, and 20 (27.39%) had multiple grooves arranged in an irregular, circinate pattern (Figure 1).

## DISCUSSION

The prevalence of fissured tongue was 73 (13.51%) in our study. The prevalence of fissured tongue worldwide varies by geographic location and has been reported to range from 0.6% (in South Africa) to 27.7% (in Brazil).^5^ Recent studies done in India on the prevalence of fissured tongue reported 28.6% and 13.0%.^3^'^6^ Studies from Afghanistan on fissured tongue reported a high prevalence of 27.2% and 35.5%.^1,7^

In our study fissure tongue was more prevalent among women than in men. This is similar to the findings of studies done in the Brazilian and Hungarian populations.^8,9^ Many studies supported a difference in prevalence between the sexes, with a higher frequency among males.^5,10,11^

A fissured tongue is a variant of normal anatomy in which several grooves appear on the dorsal surface of the tongue in numerous arrangements. These grooves can harbour candidal hyphae since cleaning them manually is not an easy task achieved by the patient. This in turn can cause soreness and discomfort to the patient. Therefore recognition of their pattern is also needed.^12^

A recent study portrayed a newer classification for the fissured tongue, based on its pattern, regularities of pattern, associated signs, and other already present systemic diseases.^12^ Studies done in Andkhoy City, Afghanistan reported the most common type of fissured tongue was the central longitudinal pattern (42.9%), whereas the lateral longitudinal pattern was the least tongue-fissured type in the study sample (8.4%).^1^

In our study, a single prominent median groove fissure pattern was most seen followed by a median groove with accessory grooves radiating laterally at right angles to the median groove. A similar finding was reported by Guntur, an Indian study wherein they reported a central longitudinal pattern in 196 (50.6%) subjects followed by a branching pattern in 68 (17.6%) subjects.^12^

A study done in Kabul reported multiple nonconnected fissure patterns to be in the majority in their study population followed by multiple connected fissure patterns and then single fissures of varying depths which is the opposite of our findings.^7^ As observed by an Afganisthan study such variations in the frequency and presentation of the fissured tongue could be characteristic of differences in the attributes of participants, community, and ethnicity in the studies sampled.^1^

The limitation of this study is that the data presented here reflects only the specific patient population reported to a single institution and not the community as a whole. Also, the sampling technique was convenience sampling which may have resulted in selection bias. Multicentre studies with a probability sampling method are recommended for future studies.

## CONCLUSIONS

The prevalence of fissured tongue among patients visiting the Department of Oral Medicine and Radiology was higher than other studies done in similar settings. There is a need to study the prevalence of fissured tongue and tongue anomalies to spread awareness as such and of its association with patients' symptoms and syndromes.
